# Introducing personalised risk based intervals in screening for diabetic retinopathy: development, implementation and assessment of safety, cost-effectiveness and patient experience (ISDR): a case study in the use of automated systems in trials

**DOI:** 10.1186/1745-6215-16-S2-O59

**Published:** 2015-11-16

**Authors:** Duncan Appelbe, Deborah Broadbent, Mehrdad Mobayen-Rahni, Antonio Eleuteri, Abigail Bennett, Tracy Moitt, Amu Wang, Marta García-Fiñana, Anthony Fisher, Simon Harding

**Affiliations:** 1 Clinical Trials Research Centre, The University of Liverpool, Liverpool, UK; 2 Department of Eye and Vision Science, University of Liverpool, Liverpool, UK; 3 St Paul’s Eye Unit, Royal Liverpool University Hospital, Liverpool, UK; 4 Department of Clinical Engineering, Royal Liverpool University Hospital, Liverpool, UK; 5 Department of Biostatistics, University of Liverpool, Liverpool, UK

## 

ISDR is a 5 year NIHR funded programme of applied research aiming to introduce a step-change in screening for sight threatening diabetic retinopathy utilising personalised risk based intervals. The research programme includes a Randomised Controlled Trial (RCT) to assess the validity of these intervals.

The RCT aims to recruit 4400 participants from a pool of 18,000 subjects in Liverpool. Due to the size of the cohort and that much of the data required for the study is collected routinely in multiple NHS organisations, the RCT relies heavily on the integration of multiple databases/data sources and automated systems across multiple networks to reduce the workload on the study team and medical practitioners and to maintain data quality.

Data for the RCT is obtained from the following systems: EMIS Web (GP Data), OptoMize (screening software provided by Digital Healthcare), OpenClinica (study database), randomisation system (bespoke system), risk engine (a bespoke application to calculate personalised screening intervals) and a record of those subjects who have consented for their data to be used by the ISDR programme.

This paper will present the processes and solutions undertaken to automate, test and validate the import/export of data, create bespoke systems to clean these data and integrate the different data sources. Thereby ensuring the timely transfer and format of data minimise the disruption to patient care while ensuring integrity of the trials data.

The views expressed are those of the authors and not necessarily those of the NHS, the NIHR or the Department of Health.

